# Hernioscopy in Incarcerated Inguinal Hernia Spontaneously Reduced after General Anesthesia Induction

**DOI:** 10.7759/cureus.1849

**Published:** 2017-11-15

**Authors:** Irwin C White-Gittens, Aleksandr Kalabin, Vishnu R Mani, Anant Dinesh, Raja Sabbagh

**Affiliations:** 1 Department of General Surgery, Columbia University College of Physicians and Surgeons at Harlem Hospital Center; 2 Department of Surgery, New York University School of Medicine, and the Laura and Isaac Perlmutter Cancer Center, Columbia University School of Physicians and Surgeons at Harlem Hospital Center

**Keywords:** incarcerated hernia, inguinal hernia, bowel necrosis, laparoscopy, hernioscopy

## Abstract

Hernioscopy is essentially hernia sac laparoscopy. Hernia repair has evolved over the years with better outcomes; however, strangulated inguinal hernias are acute surgical emergencies which require emergent operative intervention. During anesthesia induction and/or after incision, hernia self-reduction is possible, with or without compromised bowel, back into the abdominal cavity. It is pivotal to examine the bowel to decide on further operative course. A simple alternative to unnecessary laparotomy or standard laparoscopy is hernioscopy, which is quite uncommon. We present a case of an acute symptomatic strangulated left-sided inguinal hernia which got self-reduced during anesthesia induction and was successfully repaired after hernioscopy was used to evaluate the incarcerated hernia content. We provide a brief review of literature about hernioscopy and an algorithm to guide surgeons in emergent cases.

## Introduction

Progressive advances in surgical techniques have made it possible to utilize the laparoscopic approach in a great variety of elective and emergency surgical cases. The number of patients undergoing laparoscopic surgery, and the physicians trained to perform it, are constantly increasing. It is well documented that minimally invasive surgery has its own perks and advantages over open surgery like reducing surgical site infection, postoperative pain, and morbidity, in addition to shorter hospital stay and better cosmetic outcomes.

Inguinal hernia repair remains one of the most common surgeries performed in the US [1]. When patients present with symptoms and signs of incarcerated inguinal hernia, management strategies may vary but the goal of emergent repair is to alleviate bowel obstruction, remove devitalized tissue, and mitigate the risk of abdominal catastrophe.

Spontaneous reduction of an inguinal hernia is always a possibility and could represent a dilemma whether to proceed with exploratory laparotomy or laparoscopically evaluate intraabdominal contents. As no clear guidelines are currently present on the best method to rule out bowel necrosis and to assess its extent, exploratory laparotomy is usually performed at the surgeon's discretion and is associated with a higher rate of occurrence of major complications compared with the laparoscopic approach.

In our limited review of the literature, we noted innovative practices of utilizing minimally invasive approaches for specimen extraction via natural and pathological orifices [2]. A similar concept was applied to incarcerated hernia management more recently. Though the surgical mindset usually shifts towards exploratory laparotomy when suspected incarceration has spontaneously reduced, which can increase morbidity, we instead opted for a less invasive diagnostic technique and the consequent definitive surgical procedure titrated accordingly. We here present a case of an incarcerated left inguinal hernia that spontaneously reduced in the operating room and we were not able to rule out necrotic bowel. It was decided to proceed with hernioscopy, a relatively unpopular technique that we propose should be in the surgeon’s armamentarium.

## Case presentation

A 57-year-old man with past surgical history of exploratory laparotomy after a motor vehicle accident 35 years ago and past medical history of asymptomatic left inguinal hernia for the past one year presented to the emergency department with complaints of left scrotal swelling, nausea, vomiting, and obstipation for six hours. On examination, he had a large, tender, non-reducible left inguinal hernia associated with abdominal distension. Vital signs were within normal limits.

**Figure 1:**
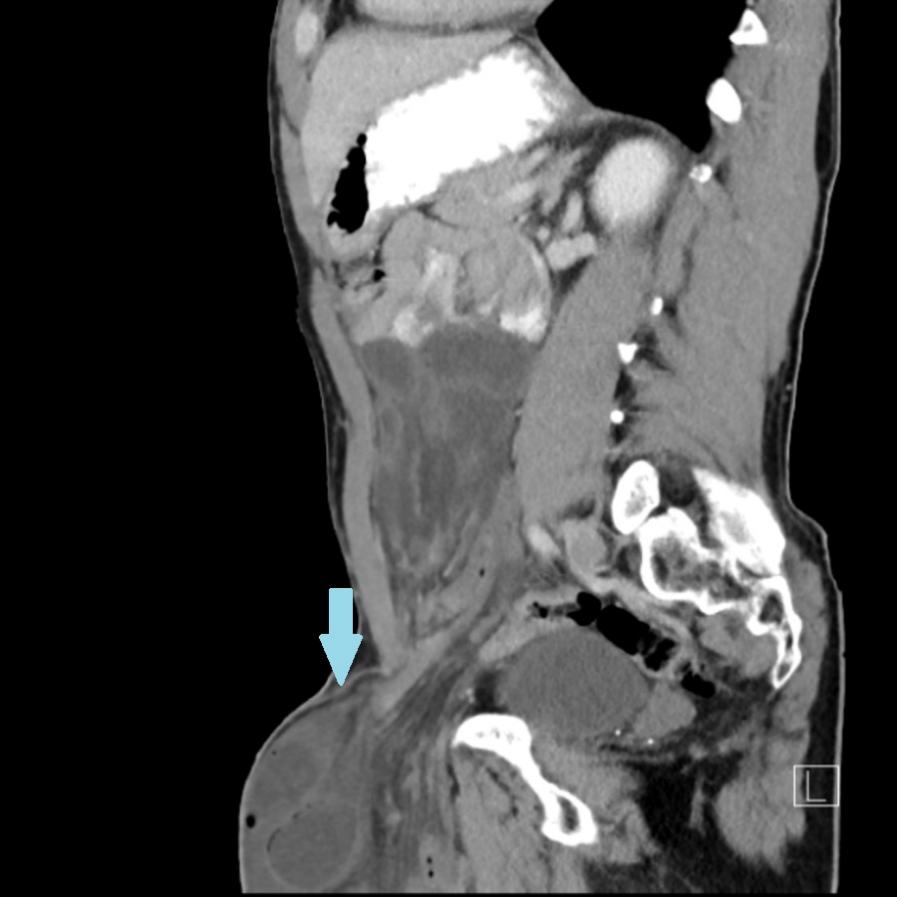
Abdominal computed tomography, sagittal plane Left inguinal hernia (arrow) contains dilated small bowel loops. Infiltration of fat and engorgement of herniated vessels and fluid are also noted.

**Figure 2:**
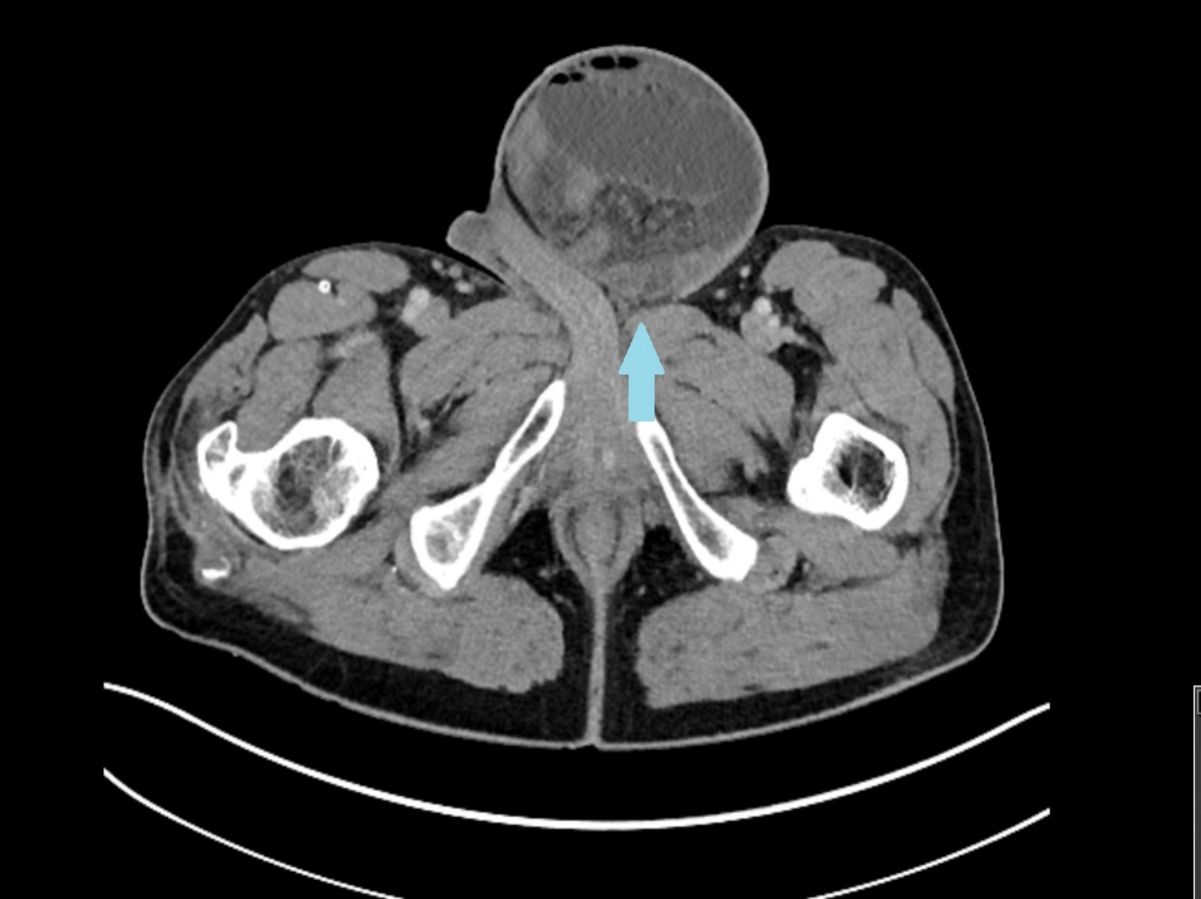
Abdominal computed tomography, axial plane Left inguinal hernia with narrow neck (arrow) contains dilated small bowel loops. Infiltration of fat, engorgement of herniated vessels and fluid are also noted.

Laboratory workup showed that his lactate level was elevated to 3.3 mmol/L and the rest of the tests were unremarkable. White blood cell count was 9.5 k/uL, hemoglobin 15.8 g/dL, and hematocrit 49.4%. A computed tomography (CT) scan with oral and intravenous contrast was obtained, which suggested ascites and a large left inguinal hernia with multiple dilated loops of small bowel within the hernia sac and collapsed loops distally with a transition point at the neck of the hernia (Figures 1-2).

**Figure 3:**
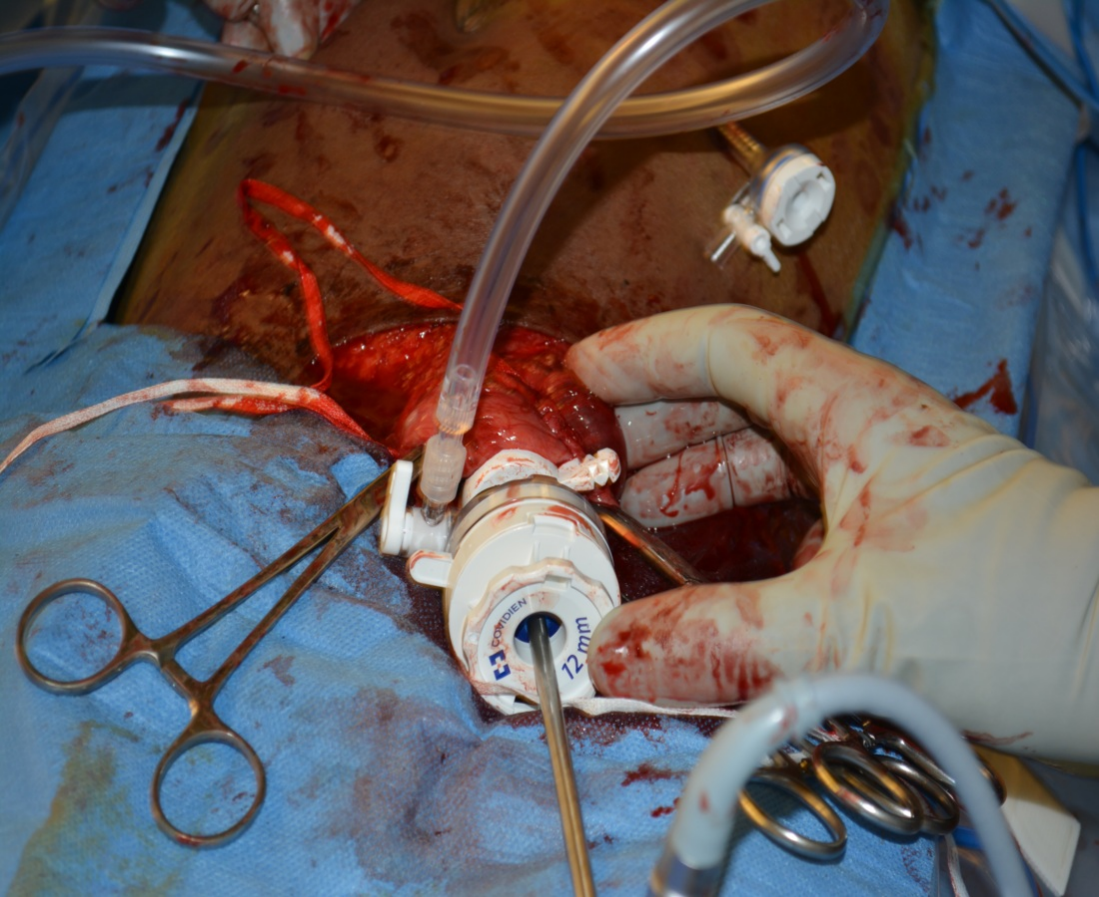
Hernioscopy 12mm trocar is placed through the hernia sac and pneumoperitoneum established. Additional 5 mm trocar is placed under direct visualization.

The patient was aggressively resuscitated with intravenous fluids and was taken to the operating room emergently for left incarcerated inguinal hernia exploration and repair. Following induction of anesthesia and groin incision, the patient’s hernia spontaneously reduced, thus precluding assessment of the viability of the bowel. For a complete bowel assessment and to rule out any ischemic changes, we decided to proceed with diagnostic laparoscopy through the hernia sac (hernioscopy) using a 12 mm trocar and a 30-degree angled laparoscope. The 12 mm trocar was placed through the inguinal hernia sac and through the deep inguinal ring into the peritoneal cavity and secured with umbilical tape (Figure 3).

A pneumo-peritoneum was successfully created and maintained. Two 5 mm trocars were then placed under direct vision in the left lower quadrant, and an assistant port in the right lower quadrant. The peritoneal cavity, including the small intestines and viscera, was examined in its entirety using laparoscopy. Findings included a small area of small mid-jejunum and corresponding mesenteric contusion, but no evidence of ischemia or necrosis. After this, a tension-free tissue repair of the inguinal hernia was done. The patient subsequently did well and was discharged home on postoperative Day 2 following adequate pain control, diet tolerance, and return of bowel function.  He was seen in the clinic two weeks later with minimal pain, no evidence of hernia recurrence, and continued bowel function.

## Discussion

The practice of hernia repair has increased tremendously owing to the number of patient surgically treated annually. Hernia surgeries are one of the most commonly performed operations worldwide, with more than 800,000 inguinal hernias alone repaired annually in the US [3]. The majority of these surgeries are performed electively with relatively low morbidity and operative mortality. The goals for elective herniorrhaphy is to perform long-lasting abdominal wall closure and prevent recurrence and feared hernia complications such as incarceration. Despite universally accepted recommendations for elective asymptomatic inguinal hernia repair [4], many patients still present to the emergency room with incarceration [5].

An incarcerated inguinal hernia is always an emergency and an indication for immediate surgery [6]. Once incarcerated, the morbidity and mortality is increased compared with an elective repair, and the goals of care are to acutely resuscitate the patient and remove all nonviable tissue and to perform a repair feasible under the circumstances as to contamination status, location, and size of the defect.

Spontaneous reduction of an incarcerated inguinal hernia without definitive evidence of viable content is a well-known problem surgeons may encounter. This could happen spontaneously in the operating room during induction of anesthesia or when an initial groin incision is already performed, and it is important to evaluate abdominal contents to prevent a disaster. However, there are no guidelines on the most appropriate intervention in case of hernia reduction and possible bowel ischemia/necrosis [7]. When bowel ischemia is suspected, the intra-abdominal exploration is obligatory to definitively assess viability, and management depends on the surgeon’s preference to proceed with exploratory laparotomy or perform diagnostic laparoscopy. It has been reported that up to 15% of patients with incarcerated inguinal hernia develop bowel necrosis requiring surgical resection [8]. This implies that proper recognition of the intestinal ischemia owing to strangulation is critically important.

Exploratory laparotomy was the gold standard for evaluating strangulated content in case of hernia reduction; however, it may contribute to a longer hospital stay, later return to full ordinary activities [9], and increased overall morbidity. Moreover, other well-known complications of laparotomy that could be avoided with hernioscopy are fascial dehiscence with subsequent hernia development or evisceration, seroma/hematoma formation, ileus, and pain syndrome. In a recently published paper, 15 patients with incarcerated inguinal hernia underwent laparoscopic exploration, and only eight of them had signs of acute incarceration with subsequent reduction of incarcerated content and no bowel resection was needed [10]. G Morris-Stiff and A Hassn described the hernioscopy technique in their case study series of five patients who underwent hernioscopy for evaluation of incarcerated hernias that had reduced spontaneously prior to inspection of sac contents. Four out of five patients did not require laparotomy and one patient had an exploratory laparotomy for a non-hernia related reason.

**Figure 4:**
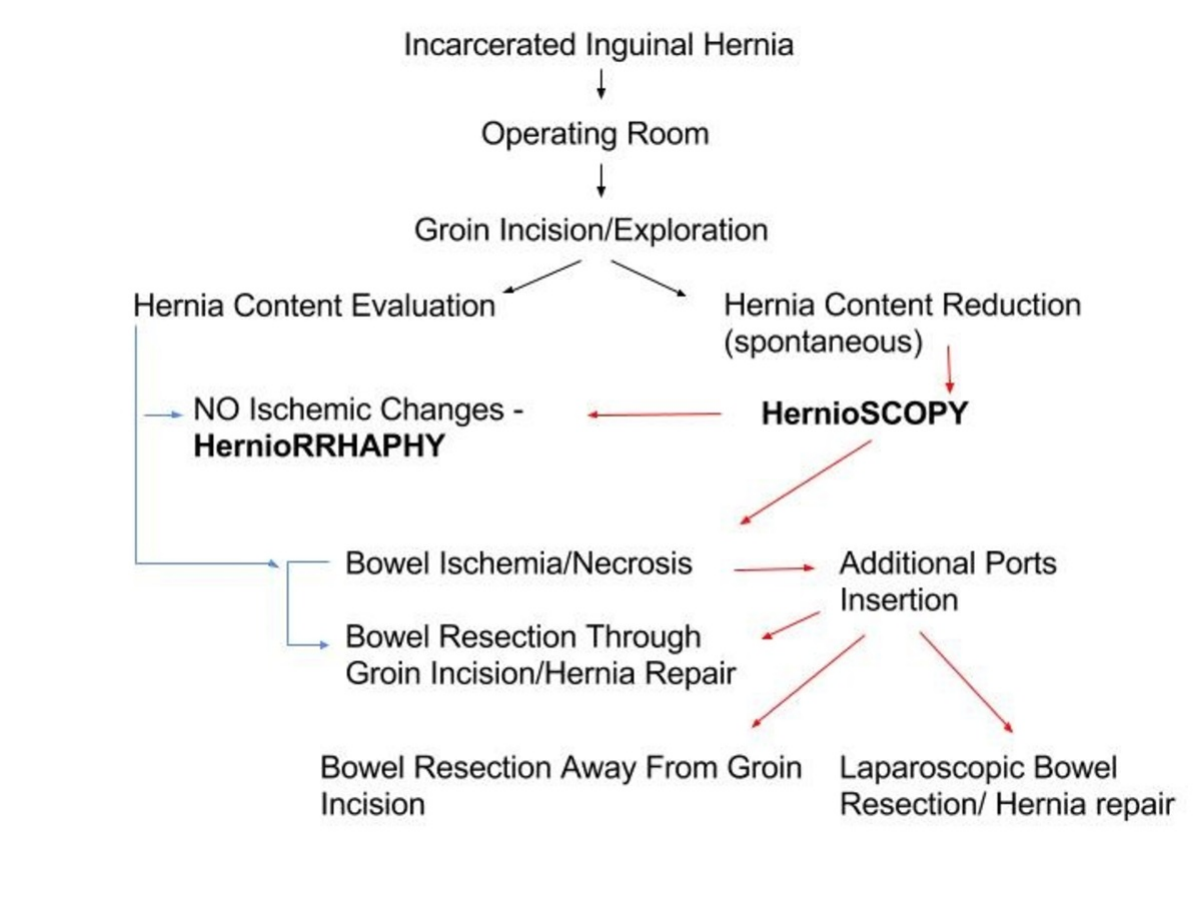
Incarcerated inguinal hernia management algorithm

As we mentioned before, with the advent of the laparoscopic approach, new techniques are emerging. Hernioscopy is a simple laparoscopy technique performed through the hernia sac. Laparoscopic assessment of bowel viability following reduction through the hernia sac is an easy and safe approach that can be employed in surgical practice especially in case of previous abdominal surgeries. The explorative potential of the technique with the current advances in laparoscopic instruments allows for a reliable assessment of the intestinal wall. Additional trocar placement could be done under direct visualization with minimized iatrogenic injury. In case of spontaneous inguinal hernia reduction, the content could be reliably observed until gradual reversal of the ischemic bowel discoloration, venous congestion, or the recovery of bowel peristalsis is confirmed, and groin incision repair is performed later in consistency with current guidelines. In case of bowel necrosis, resection could be done using the original groin incision; extension of subsequent port incisions under direct visualization could be safely done to deliver the necrotic segment away from the clean groin incision. Minilaparotomy and laparoscopic-assisted bowel resection, as well as laparoscopic resection, can relieve the patient from having a full laparotomy performed and the related complications as well as contamination of the hernia repair. Although there are no guidelines for spontaneously reduced incarcerated inguinal hernia management, we created an algorithm to help guide surgeons in emergency cases (Figure 4).

## Conclusions

Hernioscopy demonstrates a safe and simple approach for incarcerated inguinal hernia management and reduced hernia content assessment with the perspective to prevent unnecessary exploratory laparotomies and related complications with possible potential decrease in perioperative morbidity. Further management could be titrated accordingly and depends on intraoperative findings and surgeon’s experience and preference. Hernioscopy could be an accurate and reliable adjunctive diagnostic tool to expand current practices, and our algorithm could be utilized as a helpful strategy for the surgical community with the objective of minimizing the number of unnecessary exploratory laparotomies and related complications in patients with incarcerated inguinal hernia.
